# Treatment of Gonorrhea

**Published:** 1906-10

**Authors:** 


### ABSTRACTS.

### Treatment of Gonorrhea.

JOSEF RUDNIK, of Vienna, read before the 77th Congress of German Naturalists and Physicians, Sept. 24, 1905, a report of "Studies in the Treatment of Gonorrhea" (*Wiener klin. Rundschau,* No. 40, 1905). He has thoroughly tested arhovin and finds that its chief virtues are that it in no way irritates stomach or kidneys, in absolute contradistinction to the usual remedies. He cites a number of cases in illustration of its action. Many of them were seen again from time to time, examined microscopically, and found definitely cured. He concludes from his experience that arhovin is indicated in all acute and chronic gonorrheal and non-gonorrheal inflammations of the urinary passages in both sexes and all ages.

He gives internally three to six capsules each of four grains arhovin daily. For the urethra he prescribes arhovin bougies and for the vagina he orders arhovin globules. These are far easier to use for the patients than injections. Or 1% to 5% arhovin-oil solutions may be injected.

Used topically, it causes a slight burning in the inflamed mucosa, which is soon followed by an analgesia which makes urination painless. Internally, no ill effect on any organ. The discharge becomes mucoid and ceases, and ammoniacal decomposition of the urine is prevented. In this last condition arhovin is especially indicated. Sometimes the disease is aborted; more generally a rapid and painless cure occurs.

Complications and sequelæ are not entirely obviated by arhovin, but are much less frequent an account of the absence of irritation and rapidity of improvement. Epididymites are very rare; stricture is not occasioned, in fact Goldman has found the remedy of good effect on strictures when present. Cystitis and joint inflammations are rare.

He finds that arhovin has all the merits of the older antigonorrhoeics, without their drawbacks.

The remedy has also been favorably reported upon by Profs. Maramaldi , and Mosca, Drs. Piorkowski, Riess, Goldmann, Strauss, Porosz, Meissner, Steiner, Manasse, Reiner, Brings, Weger, Silberstein and others.

